# Root Reinforcement Improved Performance, Productivity, and Grain Bioactive Quality of Field-Droughted Quinoa (*Chenopodium quinoa*)

**DOI:** 10.3389/fpls.2022.860484

**Published:** 2022-03-18

**Authors:** Salma Toubali, Mohamed Ait-El-Mokhtar, Abderrahim Boutasknit, Mohamed Anli, Youssef Ait-Rahou, Wissal Benaffari, Hela Ben-Ahmed, Toshiaki Mitsui, Marouane Baslam, Abdelilah Meddich

**Affiliations:** ^1^Center of Agrobiotechnology and Bioengineering, Research Unit Labelled CNRST (Centre AgroBiotech-URL-CNRST-05), Physiology of Abiotic Stresses Team, Cadi Ayyad University, Marrakesh, Morocco; ^2^Laboratory of Agro-Food, Biotechnologies and Valorization of Plant Bioresources (AGROBIOVAL), Faculty of Science Semlalia, Cadi Ayyad University, Marrakesh, Morocco; ^3^Laboratoire Mixte Tuniso-Marocain (LMTM) de Physiologie et Biotechnologie Végétales et Changements Climatiques LPBV2C, Tunis, Tunisia; ^4^Laboratory of Biochemistry, Faculty of Agriculture, Niigata University, Niigata, Japan

**Keywords:** biostimulants, root-shoot-grain circuit, seed quality, drought tolerance, antioxidants, pseudocereal, soil health, endogenous mycorrhiza

## Abstract

Modern agriculture is facing multiple and complex challenges and has to produce more food and fiber to feed a growing population. Increasingly volatile weather and more extreme events such as droughts can reduce crop productivity. This implies the need for significant increases in production and the adoption of more efficient and sustainable production methods and adaptation to climate change. A new technological and environment-friendly management technique to improve the tolerance of quinoa grown to maturity is proposed using native microbial biostimulants (arbuscular mycorrhizal fungi; AMF) alone, in the consortium, or in combination with compost (Comp) as an organic matter source under two water treatments (normal irrigation and drought stress (DS)). Compared with controls, growth, grain yield, and all physiological traits under DS were significantly decreased while hydrogen peroxide, malondialdehyde, and antioxidative enzymatic functions were significantly increased. Under DS, biofertilizer application reverted physiological activities to normal levels and potentially strengthened quinoa’s adaptability to water shortage as compared to untreated plants. The dual combination yielded a 97% improvement in grain dry weight. Moreover, the effectiveness of microbial and compost biostimulants as a biological tool improves grain quality and limits soil degradation under DS. Elemental concentrations, particularly macronutrients, antioxidant potential (1,1-diphenyl-2-picrylhydrazyl radical scavenging activity), and bioactive compounds (phenol and flavonoid content), were accumulated at higher levels in biofertilizer-treated quinoa grain than in untreated controls. The effects of AMF + Comp on post-harvest soil fertility traits were the most positive, with significant increases in total phosphorus (47%) and organic matter (200%) content under drought conditions. Taken together, our data demonstrate that drought stress strongly influences the physiological traits, yield, and quality of quinoa. Microbial and compost biostimulation could be an effective alternative to ensure greater recovery capability, thereby maintaining relatively high levels of grain production. Our study shows that aboveground stress responses in quinoa can be modulated by signals from the microbial/compost-treated root. Further, quinoa grains are generally of higher nutritive quality when amended and inoculated with AMF as compared to non-inoculated and compost-free plants.

## Introduction

By 2050, the world’s population is expected to reach 9.7 billion, 34% higher than current levels. This will require raising overall food production by about 70% (FAO, 2017). Nearly all of this population increase will occur in many developing economies. This implies the need for significant increases in the production of several key commodities. Demand for (pseudo)cereals, for both food and animal feeds, will need to rise to some 3 billion tons, *vs* 2.1 billion today, by 2050. Major cereal crops, including wheat, rice, barley, and corn, are progressively failing to withstand the growing salinity and water scarcity present in marginal environments that are highly vulnerable to climate variability, such as shifts in growing season conditions (IPCC, 2014). Therefore, there is an urgent need to identify alternative ways to sustain and, possibly, increase agricultural production in areas where the cultivation of traditional crops has become greatly complicated and even sometimes uneconomic. Quinoa (*Chenopodium quinoa* Wild.) is an annual herbaceous pseudocereal crop originating from the Andean Mountains, where it has developed tolerance to several abiotic stresses (Di Fabio and Parraga, 2017). Quinoa grains—known as the “mother grain” of the Incan Empire—are a gluten-free alternative to starchy grains and have a low glycaemic index (Gordillo-Bastidas et al., 2016). Deemed the “Queen of Superfoods,” this plant was prized for its nutritional qualities as it contains all nine essential amino acids, carbohydrates, poly-unsaturated fatty acids, fiber, vitamins, and minerals (Ruiz-Lozano et al., 2016; Vilcacundo and Hernández-Ledesma, 2017). The grains also contain a large amount of phenolics, flavonols, and betalains (Abderrahim et al., 2015; Escribano et al., 2017). Such is the value of the plant that the UN marked 2013 as the International Year of Quinoa owing to its biodiversity and nutritional value and its role in providing food security and nutrition and poverty eradication, as well as its adaptability to thrive in a wide range of agroecosystems, as it grows at an exceptional range of altitudes, temperatures, humidities, and under different soil conditions (Bazile et al., 2013; Hussain et al., 2018).^1^ Due to its superior nutritional profile and stress tolerance, interest in quinoa is increasing worldwide, although it is still an underutilized crop with few breeding programs (Massawe et al., 2016).

More frequent and severe droughts are expected across many regions in the 21st century (Schwalm et al., 2017). Drought has a negative effect on growth, physiology, and yield (Fischer et al., 2017; Miranda-Apodaca et al., 2018). With climate change, growing alterations in the frequency, duration, and intensity of droughts and impending water disputes, the potential gains in welfare from selecting/cultivating effective drought-tolerant (DT) crops and using smart agronomical techniques, are enormous. In the past decade, total investment in drought-resistance research has clearly exceeded US$1 billion (Lybbert and Bell, 2010b). Yet, (marginal) farmers—who are typically faced with poor soils, erratic weather and limited or no access to irrigation, and other inputs—may lack the control to perceive subtle differences in the value of competing varieties and their views of the relative yield benefit of DT varieties are conditioned by (1) drought pressure: if increased cost is not associated with benefit or if the yield is comparatively lower in DT varieties than in conventional varieties when water is plentiful and (2) if the relative benefits of DT varieties fade as drought severity increases (Lybbert and Bell, 2010a). Recently, Jarvis et al. sequenced the approximately 1.5-gigabase-long genome of *C. quinoa* (Jarvis et al., 2017). Using the information gained from the genome to improve quinoa production will require robust breeding efforts and fundamental research to identify the defenses mechanism used by quinoa.

To date, manipulating a plant’s genetic makeup is a time-consuming option to enrich the new genetic variant. It is a tedious process and has several significant challenges under natural field conditions. Therefore, the demand for sustainable cropping systems has encouraged the search for technologies—from treated seeds and crop protection products to data analysis apps and precision spraying—and alternatives to increase crop production and quality/functionality with improved defense mechanisms, especially under stressful conditions. While farmer stakeholders may be able to afford to invest, small/marginal farmers have little or no access to affordable institutional credit. Further, farmers must learn how to best use these technologies to improve their farming business. To address these issues, there has been an upsurge in agricultural technologies and products. Different soil management practices that can enhance soil fertility, water availability, and the root zone could be efficient drought mitigation tools in irrigated and non-irrigated regions (Phillips et al., 2020).

In recent years, the use of plant biostimulants, either non-microbial or microbial, has become a popular option to enhance water and crop productivity and adapt crops to rapidly changing environmental conditions. The biofertilizer industry has witnessed a relatively stable market growth following the COVID-19 pandemic, with a similar trend of high demand existing in 2020. The global biofertilizer market is estimated to be worth US$ 2.6 billion in 2021 and is expected to reach a value of US$ 4.5 billion by 2026, at a compound annual growth rate (CAGR) of 12%. The need to increase food production combined with the imperative need to preserve natural resources has led to an upsurge in materials with low environmental impact but high efficiency, with natural-based substances being prioritized over synthetic inputs. The FAO recently recognized the potential of microbiomes in food production/safety, health enhancement, and environmental sustainability (FAO, 2019). Arbuscular mycorrhizal fungi (AMF) have been identified as microorganism targets that increase abiotic stress tolerance (Abdel Latef and Chaoxing, 2011). More than two-thirds of terrestrial plants acquire mineral nutrients from the soil by the root pathway *via* root epidermal cells and root hairs, and the mycorrhizal pathway (Smith and Smith, 2011). AMF recruit distinct microbes into their hyphosphere to shape the “second genome of AMF,” which significantly contributes to soil organic nutrient mobilization and turnover (Zhang et al., 2021). By colonizing the rhizosphere/endo-rhizosphere of plants, these beneficial microorganisms promote growth through various direct and indirect mechanisms (Harrison, 2005; Maillet et al., 2011). In addition, AMF application induces antioxidant enzyme activity and osmolyte accumulation under various stressful conditions, including water shortage (Meddich et al., 2018; Ben-Laouane et al., 2019, 2020; Anli et al., 2020a; Boutasknit et al., 2020b). While plants are known to establish symbiotic relationships and the role of microorganisms in managing (a)biotic stresses is gaining importance, our understanding of those relationships under a stressful environment is rudimentary. Recent research has suggested that AMF should be combined with organic amendments to increase soil fertility (Anli et al., 2020a; Ben-Laouane et al., 2020; Boutasknit et al., 2020a, 2021a; Renaut et al., 2020). Organic compost is increasingly (re)used in (modern) agriculture as a mulch to provide surface protection and it also seems to be beneficial for field crops as an alternative to chemical fertilizer overuse (Raklami et al., 2019b). It is applied to increase plant and soil health, reduce nutrient losses by volatilization or leaching, prevent soil erosion, and improve soil water retention, soil carbon content, and the long-term fertility of agricultural soils. Many stormwater manuals continue to recommend compost as an organic matter source in bioretention soils (PGC, 2014; VTSM, 2017). Quality green composts can provide nutrients and improve physical soil conditions for plant growth (Cogger, 2005). Such composts contain important concentrations of nitrogen (N), phosphorus (P), and potassium (K^+^) as well as a wide range of micronutrients, including magnesium, copper, and iron (Meddich et al., 2015).

Our growing understanding of the interconnectedness of microbiomes and organic amendment in environmental and food systems suggests that soil biofertilizer innovations have the potential to improve sustainable food, feed, and biofuel production while embracing the principles of circularity. The role of organic compounds and microorganisms in plant growth, nutrient management, and biocontrol activity is very well established. Despite the promise of biofertilizer applications, the rush to develop them should be tempered by the need to fully understand the systems in which they function and to characterize the biochemical pathways or interactions in crop systems. To the best of our knowledge, this is the first study to investigate the role of the dual application of AMF combined with compost on quinoa plants subjected to drought stress under field conditions. Moreover, quinoa seeds have potential health benefits and recent food trends and high demand have led to the adaptation and commercial production of quinoa seeds. Thus, the main objective of this research was to assess root reinforcement through a synergistic application of multiple soil biofertilizers (integrating organic amendments and native AMF) to mitigate the adverse effects of drought stress on the physiology, growth, yield, grain quality (antioxidant potential) of quinoa, and post-harvest agricultural soil fertility by regulating soil nutrient status and plant physiological processes. We hypothesized that “manipulating”/shaping the below-ground conditions in which plants grow could ultimately be beneficial. This study provides evidence toward selecting assemblages adapted to abiotic stress, improving resistance against stressors to promote the health and drought tolerance of plants.

## Materials and Methods

### Growth Conditions of Plants in the Field, Biofertilizer Material, and Collection of Vegetal Samples

Quinoa (*Chenopodium quinoa* Willd.) cv. Titicaca has been used in this study based on its high nutritional value, early-maturity standard, top performance, and extensive cultivation (high potential of adaptation in the poor organic/mineral matters) in the Andean states, Sahel, the Middle East, and North African regions (Alvar-Beltrán et al., 2020; Miller et al., 2021; Fghire et al., 2022). Quinoa seeds were surface sterilized with sodium hypochlorite solution containing 0.02% Tween 20 for 10 min. After successive washings in sterile water, the seeds were placed on 1% agar plates containing 1/2 Murashige and Skoog (1/2 MS) medium and incubated in dark conditions at 30°C for 3 days. The uniform looking seedlings were transplanted in the field at a spacing of 20 cm (same row) × 80 cm (between rows) at Saada fields, Marrakesh, Morocco (31°37′39.9″ N and 08°07′46.7″ W), during February–June 2020. The climate of this location is semi-arid, with an average annual temperature of 19.6°C and an average annual rainfall of 250 mm (from September to June). Meteorological data (minimum and maximum temperature, minimum and maximum relative humidity, wind speed, solar radiation, and rainfall) were measured by iMETOS^®^ ag weather stations installed in the field and presented in Supplementary Figure S1.

The fields have never been treated before with chemical fertilizers or other organic fertilizers. The physicochemical characteristics of the agricultural soil are reported in Table 1. All agronomic practices were performed uniformly for the quinoa plants, following the local cultural practices. We number the weeks according to the plants growing dates. Week 0 coincides with seedling emergence. Plants were subjected to two water regimes: 100% field capacity (FC; WW; well-watered regularly at 8 l/h) and 50% FC (DS; drought stress *ca*. 4 l/h) conditions applied from week 1 until harvest. The field plots were irrigated at five-day intervals using drip irrigation system lines, with appropriate internal drippers, placed on the soil surface of each furrow. The WW plots received 100% FC of the weekly calculated crop evapotranspiration for the 5 days before each irrigation. Crop evapotranspiration was determined according to Xu et al. (2018) using potential evapotranspiration multiplied by the crop coefficient, which was adjusted according to crop growth stage.

**Table 1:** Physicochemical parameters of agricultural soil and compost used.

	Soil	Compost
Sand (%) Clay (%) Loam (%)	52 24 24	
pH	7.90 ± 0.07	7.74 ± 0.01
EC (mS cm^−2^)	1.70 ± 0.60	5.46 ± 0.20
TKN (%)	0.15 ± 0.01	1.32 ± 0.01
${\mathrm{NH}}_{4}^{+}$ (%)	_	0.09 ± 0.01
NO_3_^−^ (%)	_	0.31 ± 0.01
TOC (%)	1.30 ± 0.30	5.72 ± 0.45
OM (%)	2.24 ± 0.50	9.86 ± 0.78
C/N	_	7.49 ± 0.00
${\mathrm{NH}}_{4}^{+}$ / ${\mathrm{NO}}_{3}^{-}$	_	0.29 ± 0.00
Polsen (ppm)	31.00 ± 2.00	489.95 ± 20.3
Na^+^ (mg g^−1^)	1.24 ± 0.08	2.11 ± 0.05
K^+^ (mg g^−1^)	1.43 ± 0.01	5.59 ± 0.15
Ca^2+^ (mg g^−1^)	17.77 ± 2.03	37.38 ± 1.84

EC, electrical conductivity; TKN, total Kjeldahl nitrogen; TOC, total organic carbon; OM, organic matter; C/N, carbon-to-nitrogen ratio; and P, phosphorus. Values represent the mean ± standard deviation (SD).

We extracted and isolated an indigenous consortium of AMF isolated from the Tafilalet palm grove located 500 km southeast of Marrakesh (Morocco) and containing a mixture of 15 species: *Acaulospora delicata*, *A. leavis*, *Acaulospora* sp., *Claroideoglomus claroideum*, *Glomus aggregatum*, *G. claroides*, *G. clarum*, *G. deserticola*, *G. heterosporum*, *G. macrocarpum*, *G. microcarpum*, *G. versiforme*, *Glomus* sp., *Rhizophagus intraradices*, and *Pacispora boliviana* (Meddich et al., 2015). These species belong to five genera: *Glomus* (60% of the total community), *Acaulospora* (20%), *Claroideoglomus*, *Rhizophagus*, and *Pacispora* (all these three 6.7%). The number of AMF spores detected in this inoculum was 47 spores/100 g of the soil sample. The inoculum was enriched in propagules by co-cultivation with *Zea mays* L. as the host plant under controlled greenhouse conditions. Corn roots containing hyphae, vesicles, and spores were harvested, cut into small pieces and used as the inoculum. Inoculation of quinoa plants was performed by adding 2 g (/plant) of the inoculum (roots and substrate containing spores) to the quinoa root system. Non-mycorrhizal (NM) treatments received an equal quantity of both non-inoculated (and non-mycorrhizal) *Z. mays* roots to match “organic matter” in the pots and filtered inoculum in an attempt to restore other soil free-living microorganisms accompanying the AMF. The filtrate for each pot was obtained by passing the mycorrhizal inoculum in 20 ml of distilled water through a layer of 15- to 20-mm filter papers (Whatman, GE Healthcare, Buckinghamshire, United Kingdom).

The compost used in this study was prepared from green waste (quack grass) as described by Meddich et al. (2016). The physicochemical properties of the compost are presented in Table 1. The compost was applied to the corresponding plot at a rate of 1.2 Kg/plot (10 t/ha).

Each water regime (WW or DS) comprised four treatments: (1) Control: non-inoculated and non-amended, (2) AMF: seedlings inoculated with the indigenous AMF consortium, (3) Comp: seedlings amended with compost, and (4) AMF + Comp: joint application of AMF and Comp. The experimental design was carried out as a split-plot based on a randomized complete block design with three replications. The fields were divided into 24 plots, 0.8 m wide separated from each other by 0.5 m; of six rows each (∼ 60 plants per plot). Each plot was randomly assigned a watering regime (WW or DS) comprising biofertilizers (or not) treatments, with three replicates, for a total of 24 (2×4×3).

### Harvesting, Plant Growth, and Yield Parameters

After 4 months of cultivation, at the harvest time and within each plot, individual plant samples were manually collected at the same time of the day (10 am to 1 pm). Ten representative plant samples from each plot were collected. Roots were collected using a shovel from 30 cm depth of each plant’s system, avoiding brace roots, cutting using garden shears, and kept into an aluminum foil bag and then pooled. Roots were vortexed for 2 min in epiphyte removal buffer to remove rhizosphere soil, rinsed twice in root washing buffer, gently wiped, and placed in aluminum foil bags for the subsequent analyses.

The growth performance of quinoa plants was assessed by measuring the seed yield, shoot height (SH), root elongation (RE), biomass production (PDM; plant dry matter), and seeds dry matter (SDM). DM was obtained after drying samples at 80°C until the weight remained constant. Seed samples were cleaned and stored (4°C) for subsequent analyses. The tolerance index (TI) was calculated using the DMs according to the methods described by Parvez et al. (2020) using the following formula:


$$\mathrm{Tolerance index}\;\left(\mathrm{TI}\right)\;\left(\%\right)=\left(\frac{\mathrm{DMstressed plants}}{\mathrm{DMwell}-\mathrm{watered}}\right)\times 100$$

### Mycorrhization Assessement

The harvested roots were washed with distilled water and cleared with 10% KOH at 90°C for 30 min. Then, they were rewashed and acidified with 2% HCl for 10 min and stained with Trypan blue at 90°C for 20 min, according to Phillips and Hayman (1970). Root fragments of 1 cm long were observed in the glycerol droplet. The microscopic assessment of mycorrhizal root colonization rates (F %: frequency and I %: intensity) was performed according to the method of Derkowska et al. (2008) using 20 randomly selected root fragments repeated five times for each sample.


$$\mathrm{Mycorrhizal frequency}\;\left(F\right)\;\left(\%\right)=\left(\frac{\mathrm{Infected root segments}}{\mathrm{Total roots segments}}\right)\times 100$$

$$\mathrm{Mycorrhizal intensity}\;\left(I\right)\;\left(\%\right)=\frac{\left(95n5+70n4+30n3+5n2+n1\right)}{\mathrm{Total roots segments}}$$

Where n represents fragments with an index of 0, 1, 2, 3, 4, or 5 with the following infection rates: 100 > n5 > 90; 90 > n4 > 50; 50 > n3 > 10; 10 > n2 > 1; and 1 > n1 > 0.

### Grain Mineral Composition Analysis

Mineral concentrations (K, Ca, and Na) in grain were determined after digestion using ICP/OES Ultima Expert (inductively coupled plasma/optical emission spectrometry, iCAP 6500 Duo, Horiba Inc., Burlington, ON, Canada). The total phosphorus in grains was determined according to the method described by Olsen and Sommers (1982).

### Measurement of Chlorophyll and Carotenoids Concentration

The concentration of chlorophyll *a*, *b*, total chlorophyll, and carotenoids was determined using the method of Lichtenthaler (1987). Leaf tissue (100 mg) was homogenized in 80% pre-chilled acetone. After centrifugation at 10,000 *× g* for 10 min, the supernatants were pooled and the absorbance of the extract was read at 480, 645, and 663 nm using a UV/visible spectrophotometer (UV-3100 PC spectrophotometer).

### Total Soluble Sugars and Protein Quantification

The quantity of total soluble sugars (TSS) content was determined according to Kochert (1978) in 0.25 ml of the supernatant mixed with 0.25 ml of phenol and 1.25 ml of sulfuric acid. After 15 min, TSS content was determined by measuring the absorbance at 485 nm and calculated using the standard glucose curve.

Total soluble proteins were determined according to the technique described by Bradford (1976). Samples (1 g) were homogenized with 4 ml of 1 M phosphate buffer (pH 7.2) and then centrifuged at 18,000 *x g* for 15 min at 4°C. The absorbance was read at 595 nm.

### Stress Indicators (Malondialdehyde and Hydrogen Peroxide) Determination

Hydrogen peroxide (H_2_O_2_) concentration in seeds and leaves was determined by the method described by Velikova et al. (2000). Briefly, samples were homogenized with 5 ml 10% (w/v). Trichloroacetic acid (TCA) in an ice bath and then centrifuged at 12,000 × *g* for 10 min at 4°C. The supernatant (0.5 ml) was recovered to determine the content of H_2_O_2_ and 0.5 ml of potassium phosphate buffer (10 mM, pH 7) and 1 ml of iodic potassium (1 M) was added. After 1 h of incubation, the absorbance at 390 nm was recorded and plotted against a standard H_2_O_2_ curve. The blank was made by replacing the sample extract with 10% TCA.

Lipid peroxidation as malondialdehyde (MDA) equivalent was evaluated in seeds and leaves tissues. MDA content was estimated by seed samples (0.1 g) in 3 ml of 0.1% (w/v) TCA and centrifuged at 18,000 × *g* for 10 min as described by Rao and Sresty (2000). Supernatant was mixed with 3 ml of 0.1% TCA containing 0.5% (w/v) thiobarbituric acid (TBA). The mixture was then heated in a water bath at 100°C for 30 min and immediately cooled in an ice bath. The absorbance was read at 440, 532, and 600 nm. The concentration of MDA (nmol g^−1^ DW) was calculated by using the extinction coefficient of 155 mM cm^−1^, and the results were expressed as nmol MDA equivalents per grams.

### Preparation of Methanolic Extracts

Two grams of ground quinoa seeds was mixed with 50 ml of 80% (v/v) methanol at room temperature with frequent agitation. The mixtures were then left in a shaking incubator prior to filtration (Buckner funnel and Whatman No. 1) for 24 h and were then centrifuged at 3,500 *× g* for 10 min at 5°C. The clarified extract was collected and then evaporated to dryness using a rotary evaporator at 40°C. Finally, the extract was reconstituted in sterile distilled water, freeze-dried, and stored at 4°C in an airtight container until further use.

### Determination of Total Phenols and Flavonoids Content and 1,1-Diphenyl-2-Picrylhydrazyl Radical Scavenging Assay in Quinoa Grain

The total phenolic content (TPC) was measured using the Singleton et al. (1999) assay with slight modifications. To determine the total flavonoids, gallic acid standard was used to prepare the calibration curve. Briefly, a 250 μl aliquot of appropriately diluted methanolic extract solution (with distilled water) was placed in a test tube, to which 2.5 ml of 1 N Folin–Ciocalteu reagent solution was added. The mixture was incubated at room temperature for 3 min. Then, 250 μl of 10% sodium carbonate (Na_2_CO_3_) solution was added and kept in a dark place for 90 min. The absorbance of the extract solution and different concentrations of quercetin standard was measured at 760 nm. The TPC was calculated using an established formula and was expressed as milligrams of gallic acid equivalents (GAE) per g of quinoa sample DM (mg GAE/g DM).

The total flavonoids content (TFC) was determined using the colorimetric method described by Li et al. (2006). A quercetin standard was used to prepare the calibration standard curve to determine the TFC. A 0.5 ml aliquot of appropriately diluted methanol extract solution was taken in a test tube, to which 0.3 ml of 5% sodium nitrate solution was added. The mixture was kept at room temperature for 5 min. Then, 0.3 ml of 10% aluminum nitrate was added to each test tube, kept in a dark place for 6 min, and the reaction was stopped by adding 2 ml of 1 M sodium hydroxide. The absorbance of the extract solution and different concentrations of quercetin standard were measured at 510 nm. The standard calibration curve was prepared by plotting the concentration *vs* quercetin absorbance. Finally, The TFC was calculated using an established formula and was expressed as mg of quercetin equivalents.

The free radical scavenging activity of the quinoa seeds was assessed using the modified 1,1-diphenyl-2-picrylhydrazyl (DPPH) radical scavenging assays of Athamena et al. (2010). A 50 μl aliquot of appropriately diluted methanol extract solution was placed in a test tube, to which 2 ml of freshly prepared 0.1 mM DPPH methanol solution was added. An equal amount of methanol was used as a control. After vigorously shaken incubation for 20 min in a dark place at room temperature, the absorbance of the extract solution and different concentrations of ascorbic acid standard were measured at 517 nm. The scavenging activity was calculated using the following formula:


$$\mathrm{DPPH radical scavenging}\;\left(\%\right)=\left(\frac{\mathrm{Acontrol}-\mathrm{Asample}}{\mathrm{Acontrol}}\right)\times 100$$

The IC50 (half-maximal inhibitory concentration) value was also calculated for the dose inhibition curve in linear range by plotting the extract concentration vs. the corresponding scavenging effect.

### Antioxidant Enzymes Activities of Quinoa

Ground samples (100 mg) were mixed with 4 ml of 0.1 M sodium phosphate buffer (pH 7) containing 5% insoluble polyvinylpyrrolidone (PVPP) and 0.1 mM ethylene diamine tetraacetic acid (EDTA). The homogenate was centrifuged at 18,000 *× g* for 10 min at 4°C, and the supernatant was collected and used to measure antioxidant enzymes activities. Total soluble proteins were determined according to the technique described by Bradford (1976).

*Superoxide dismutase* (*SOD, EC1.15.1.1*) activity was assayed using the method of Beyer and Fridovich (1987). One unit of SOD activity was defined as the amount of enzyme leading to 50% inhibition of nitro blue tetrazolium (NBT) reduction at 25°C. The SOD activity was expressed at unit mg protein^−1^ min^−1^.

*Ascorbate peroxidase* (*APX, EC1.11.1.11*) activity was measured according to the method of Amako et al. (1994). APX was assayed as a decrease in absorbance at 290 nm for 1 min. The assay mixture contained 100 μl of extract sample, 50 mM potassium phosphate buffer (pH 7.6) including 0.2 mM EDTA, 100 μM H_2_O_2_, and 0.5 mM ascorbate. The reaction was initiated by adding the enzyme extract, and the decrease in absorbance was recorded. One enzyme unit was defined as μmol mg^−1^ protein oxidized ascorbate per min.

*Peroxidase* (*POX, EC 1.11.1.7*) activity was measured using the guaiacol test by monitoring the change of absorbance at 470 nm. The activity was assayed for 3 min in a reaction solution containing 3 ml of 1 M potassium phosphate buffer (pH 7.0), 20 mM guaiacol, 40 mM H_2_O_2_, and 0.1 ml enzyme extract in a 3 ml volume (Polle et al., 1994). POX activity was determined by its ability to convert guaiacol to tetraguaiacol (*ε* = 26.6 mM^−1^ cm^−1^). One unit of POX activity was defined as an absorbance change of 0.01 unit min^−1^.

*Polyphenol oxidase* (*PPO, EC 1.14.18.1*) activity was estimated by the method of Hori et al. (1997). The assay solution contained 20 mM catechol in 0.1 M phosphate buffer (pH 7). The reaction was started by adding 100 ml of the enzymatic extract. PPO activity was expressed in enzyme unit mg^−1^ protein. One unit of PPO activity was defined as the amount of enzyme causing an increase in the absorbance of 0.001/min at 420 nm.

### Soil Analyses

The physicochemical properties of agricultural field soil were analyzed at plant harvest on samples taken near the roots to assess the effect of compost and/or AMF applied alone or in combination with soil fertility. Five homogeneous rhizospheric soil samples at a 0–20 cm depth were collected for each treatment. The samples were dried and sieved to measure the pH, electrical conductivity (EC), total organic carbon (TOC), organic matter (OM), and assimilable phosphorus (AP). The pH and EC were measured in a diluted soil suspension 1/5 (v/v) using a pH meter HI 9025 and a conductivity meter HI-9033 (Hanna Instruments, Padua, Italy), respectively. Total TOC and OM were measured according to the method described by Aubert (1978), which consists of the oxidation of organic matter by potassium dichromate in the presence of sulfuric acid. The AP concentration was determined according to Olsen and Sommers (1982).

### Statistical Analysis

Statistical analysis was carried out with the software package CoStat version 6.400 (CoHort Software). Data were analyzed by employing a three-way multivariate analysis of variance (MANOVA) followed by the Student–Newman–Keuls test using a significance level of 5% (*p* ≤ 0.05). The normality of residuals was tested using the Shapiro–Wilk test. Mycorrhizal root colonization rates were arcsin-square root transformed to fit the assumption of normal distribution. Different lower cases indicate significant differences among treatments at *p* ≤ 0.05. In order to integrate all the data, a complete dataset comprising all growth, physiological, and biochemical data was subjected to Principal Components Analysis (PCA). The PCA was performed using XLSTAT v. 2016 (Addinsoft, NY, United States). Initially, index values for each treatment were calculated by assessing the response of drought stress compared to its control value. The responses of all the traits under each treatment were combined and used as index values for PCA analysis. These index values were used to identify the correlation of response variable vectors and treatments across the ordination space. Percentage contributions of principal component (PC) variables are shown in Supplementary Table S2. The heatmap was performed using the software GraphPad® Prism v9.0 GraphPad (San Diego, CA, United States). Data are presented as mean ± standard error (SE) of three independent biological replicates.

## Results and Discussion

Drought remains one of the most damaging environmental stresses limiting crop production worldwide. Increasing food supply significantly in an unstable environment in the required timeframe is time-consuming. Hence, it is essential to find eco-friendly strategies that allow plants to withstand drought, focus on biomass and functionality, ensure food security, and mitigate the damage occurring in drought-prone areas. To our knowledge, this is the first study to summarize the extent to which the effect of single and multiple combinations of endogenous AMF and/or compost-based amendments are crucial to improve the fitness and health of quinoa—an immense industrial crop—under field drought conditions. Therefore, this method could be used for designing a functionally reliable system which could be termed “plant probiotics.”

### Effect of Drought Stress and Biofertilizers on the Root, Plant Growth, and Yield

**Figure 1:**
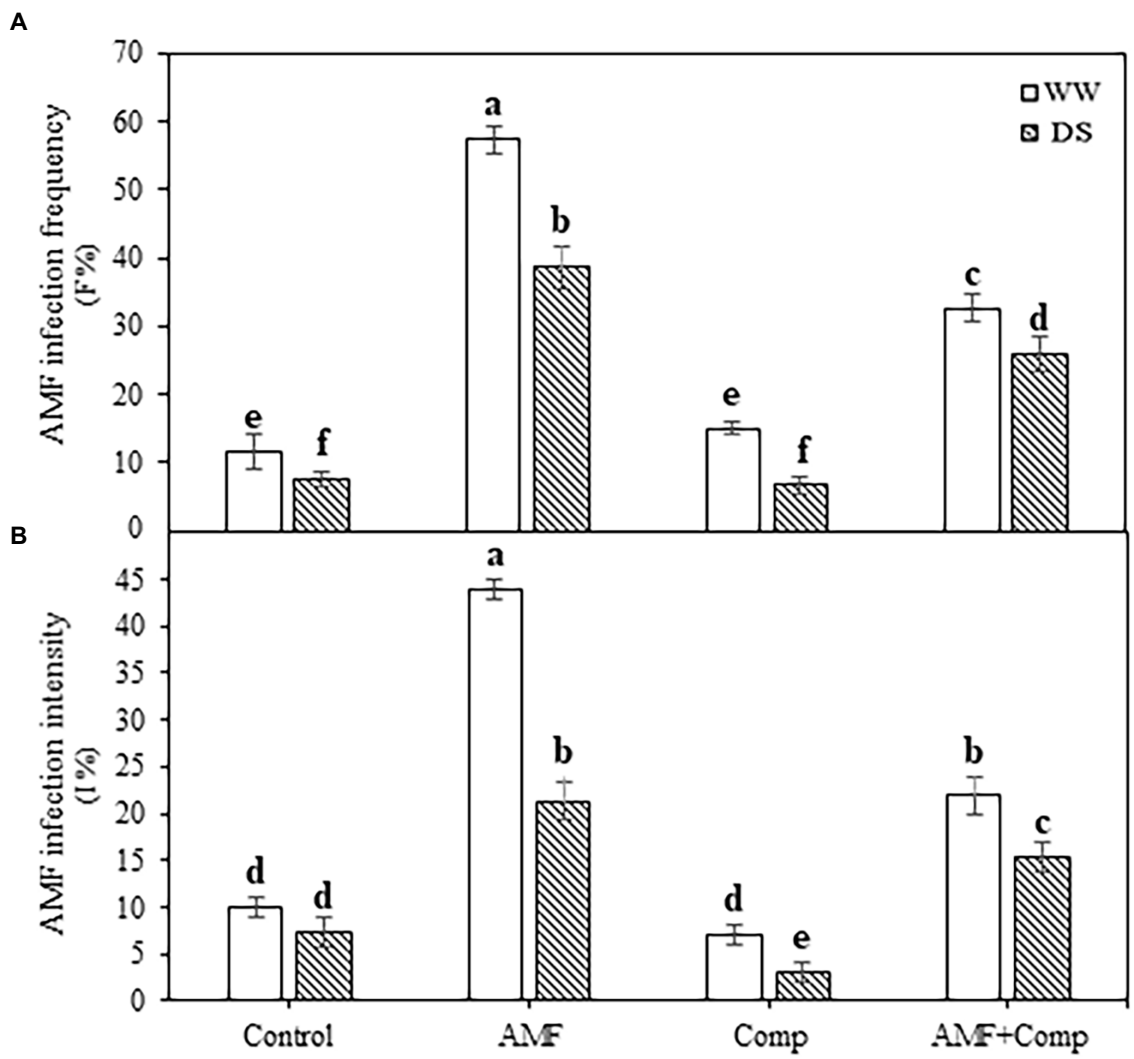
Effects of water regimes (WW; well-watered or DS: drought stress) on AMF infection **(A)** frequency and **(B)** intensity of non-amended and non-inoculated quinoa (control) and quinoa inoculated with arbuscular mycorrhizal fungi (AMF) and/or amended with compost (Comp). Data are mean ± SD. Means followed by the same letters are not significantly different at *p* < 0.05.

The AMF infection frequency and intensity were estimated in the root system of quinoa plants (Figure 1). Under field conditions, the non-inoculated plants exhibited mycorrhizal infections with or without stress. The addition of AMF alone successfully infected quinoa roots and showed the highest root mycorrhizal frequency (57%) and intensity (44%) regardless of the water regime. The application of drought stress significantly (*p* < 0.001, Supplementary Table S1) reduced mycorrhizal frequency and intensity in all the treatments. The negative effect of drought on the AMF colonization of quinoa plants is in line with several studies showing that mycorrhizal infection decreased in drought-treated host plants (Baslam and Goicoechea, 2012; Meddich et al., 2015; Anli et al., 2020a). Drought induces inhibition of AMF spore germination and hyphal growth, thereby decreasing mycorrhizal colonization (Rydlová and Püschel, 2020). In addition, soil moisture plays a vital role in spore germination and/or development (Püschel et al., 2021). The supply of compost significantly (*p* < 0.001, Supplementary Table S1) decreased mycorrhizal frequency by *ca*. 40 and 30% and intensity by *ca*. 50 and 30% in AMF-inoculated plants under non-stressed and stressed conditions, respectively. The application of organic amendments including compost and mineral nutrients may reduce AMF root colonization and activity owing to the release of mineralized P in the soil and/or diffusion of decomposition products, which reduces the establishment and maintenance of the symbioses (Gryndler et al., 2008; Baslam et al., 2011). Other studies, in contrast, have revealed that compost application could have a positive effect on AMF growth and sporulation (Cavagnaro et al., 2015; Anli et al., 2020a,b). There may be several reasons for this favorable effect of compost on AMF; N- and humic acid-rich compost (Gryndler et al., 2009), moderate level of soil assimilable P (Yang et al., 2017), and/or compost with no AM fungal spores that could outcompete the native microbial community once the compost is applied to the soil (Yang et al., 2018).

Several studies have been conducted which demonstrate the beneficial effects of AMF and/or compost on plant performance. Here, we also showed that mycorrhizal symbiosis in the presence or absence of compost improved quinoa biomass and grain quality. In addition to the lower AMF colonization levels under drought stress, we observed higher growth traits in the treated plants relative to those in the control non-amended and non-inoculated plants. These results indicate that compost supply and AMF colonization improved the growth of treated plants. Drought stress jeopardizes plant growth and causes a series of physiological, biochemical, and molecular changes. A large number of studies have reported that biofertilizers enhance the ability of plants to tolerate drought stress through multiple mechanisms in leaves and below-ground, including soil structure improvement, increased formation of soil aggregates, and increases in soil nutrient and moisture retention. Plant nutrient and water absorption have been found to be accelerated due to the vast networks of extraradical mycelium, plant photosynthetic, and defense mechanisms which alleviate harmful reactive oxygen species (ROS) effects have been found to be improved and increases in the expression of drought-resistance genes have been observed (Mo et al., 2016). Here, we were interested in determining whether biofertilizer application would improve quinoa grain traits under drought stress.

The deleterious effect of water stress was observed on all growth parameters (shoot height, root elongation, and plant and grain dry weights) and quinoa yield cultivated under field conditions (Table 2). The results revealed that the growth traits and yield of plants were significantly (*p* < 0.001, Supplementary Table S1) reduced under water scarcity. However, biofertilizer application mitigated the negative effect of water deficit and improved the growth of quinoa as compared to the controls. The application of compost and AMF, separately or in combination, significantly increased plant biomass under stressed and non-stressed conditions compared to non-inoculated and non-amended plants. In fact, the highest plant growth values were obtained when the compost was combined with AMF (AMF + Comp). Under DS conditions, the AMF + Comp treatment increased plant dry weight (*ca*. 200%), grain dry weight (*ca*. 100%), shoot height (*ca*. 70%), root elongation (32%), and yield (*ca*. 100%) in comparison with stressed control plants.

**Table 2:** Growth performance, yield, and tolerance index of non-amended and non-inoculated quinoa (control), and quinoa inoculated with arbuscular mycorrhizal fungi (AMF) and/or amended with compost (Comp) subjected to different water regimes (WW; well-watered or DS: drought stress).

		Shoot height	Root elongation	Plant dry weight	Grain dry weight	Yield
		(cm)	(cm)	(g/plant)	(g/plant)	(tons/ha)
WW	Control	97.3 ± 2.5^c^	44.2 ± 0.7^de^	5.7 ± 0.2^e^	15.6 ± 0.7^d^	3.9 ± 0.2^d^
	AMF	116.7 ± 2.9^b^	53.7 ± 1.5^b^	18.0 ± 1.3^b^	24.0 ± 1.0^b^	6.0 ± 0.3^b^
	Comp	110.3 ± 0.6^b^	43.3 ± 1.4^e^	14.9 ± 0.6^c^	21.5 ± 0.5^c^	5.4 ± 0.1
	AMF + Comp	123.3 ± 3.0^a^	57.3 ± 1.4^a^	21.6 ± 0.5^a^	32.0 ± 2.6^a^	8.0 ± 0.7^a^
DS	Control	77.3 ± 2.9^d^	35.5 ± 0.9^g^	4.4 ± 0.4^f^	7.9 ± 1.4^f^	1.9 ± 0.3^f^
	AMF	112.0 ± 1.7^b^	45.9 ± 1.9^cd^	9.5 ± 0.5^d^	15.0 ± 1.0^d^	3.8 ± 0.2^d^
	Comp	108.0 ± 7.2^b^	39.2 ± 0.8^f^	7.1 ± 0.3^e^	12.0 ± 1.0^e^	3.0 ± 0.2^e^
	AMF + Comp	110.0 ± 4.6^b^	47.2 ± 0.7^c^	9.7 ± 0.8^d^	15.6 ± 0.5^d^	3.9 ± 0.1^d^
				**Tolerance index (%)**		
				76.72 ± 5.1		

Values represent the mean ± standard deviation (SD; *n* = 3). Means followed by the same letters are not significantly different (*p* ≤ 0.05).

The tolerance index data revealed that the quinoa variety used (Titicaca) was highly tolerant of water deficit (76%; Table 2). Our results are in agreement with previous reports (Finlay, 2008; Anli et al., 2020a), showing that AMF inoculation combined with compost application yielded the highest values of plant growth parameters. The beneficial effect of mycorrhizal symbiosis and compost application on the growth of quinoa under drought could be explained by the greater uptake of low mobility nutrients, such as P and N contained in the substrate. Several studies have indicated that compost, AMF, and PGPR improve plant growth through the assimilation of immobile soil nutrients such as N and P (Tanwar et al., 2013; Baslam et al., 2014; Abdel Latef et al., 2016; Al-Karaki, 2016; Barje et al., 2016; Frosi et al., 2016; Yang et al., 2018; Raklami et al., 2019a; Anli et al., 2021). Previous reports have demonstrated that crops inoculated with AMF accumulated more N and P in leaves than non-mycorrhizal plants when subjected to drought stress (Meddich et al., 2015). Nadeem et al. (2014) showed that AMF could regulate mineral nutrition by solubilizing soil nutrients and producing plant growth regulators (i.e., hormones). This improvement in growth traits could also be due to the growth-promoting mechanisms employed by AMF through the production of phytohormones and the solubilization of minerals (Finlay, 2008). In addition, compost is used as a soil amendment in agriculture to improve organic C supply and increase the storage capacity of water and nutrients in the soil, resulting in a higher photosynthesis rate, growth, and plant stress tolerance (Anli et al., 2020a; Boutasknit et al., 2021b). In this study, growth improvement was accompanied by better quantitative productivity. The significant boost in yield was observed in plants inoculated with AMF and amended with compost.

### Biofertilizers Enhanced Nutrient Uptake and the Concentration of Nutrients in Quinoa Grains

Cereal crops such as quinoa provide key nutritional elements to the human diet. However, abiotic stresses can negatively impact the concentration and distribution of nutrients in cereal grains. Indeed, P, K^+^, and Ca^2+^ contents were significantly (*p* < 0.001, Supplementary Table S1) decreased under water-deficit conditions. In contrast, Na^+^ uptake was significantly (*p* < 0.001, Supplementary Table S1) higher in stressed plants). Elemental concentrations, particularly macronutrients, were greater in biofertilizer-treated quinoa grain than in untreated plants (Table 3). Under water-deficiency conditions, the dual application of AMF and compost showed the most significant increase in P, K^+^, and Ca^2+^ nutrients and a maximum decrease in Na^+^ (74%) content as compared to control plants under the same condition. This was followed by the application of AMF alone, which registered a significant increase in P and Ca^2+^ uptake. Cakmak reported that biofortification of cereals by using breeding or fertilizer techniques is considered a viable way to increase the concentrations of essential nutrients in the grain (Cakmak, 2008). Several studies showed that AMF inoculation affects the yield and nutrition of crops (i.e., bread and durum wheat, barley, sorghum, legume, and other food crops; Lehmann et al., 2014; Watts-Williams and Cavagnaro, 2014; Lehmann and Rillig, 2015), which has led to AMF being proposed as one part of the solution to food security (Thirkell et al., 2017), but there has been little focus on the effects on the grain. In our study, the highest elemental macronutrients in quinoa grain in decreasing order were P, K, and Ca. Phosphorus element plays critical roles in plant function, as it is a building block of nucleic acids and phospholipids. Our data showed that AMF-colonizing quinoa root systems supplemented with compost increased the P concentration by *ca*. 10× compared to untreated plants under both WW and DS conditions. Mycorrhizae are beneficial particularly for P nutrition since the influx of P in mycorrhizal roots can be 3–5× higher than the P influx in non-mycorrhizal roots (Smith and Read, 2008). This nutrient uptake is generally improved in AMF-treated plants (Smith and Smith, 2011) and is more important when different fungi colonize plant roots. Tian et al. (2013) reported that different AMF species act differently in terms of growth, P uptake, and P transporter gene expression in maize and that simultaneous root colonization with these species induces the greatest expression of P transporters. Watts-Williams and Gilbert (2021) showed higher macronutrient P, Mg, K, and S, and micronutrient Zn concentrations in mycorrhizal barley grain than in the mock-inoculated plants.

**Table 3:** Seeds mineral composition of non-amended and non-inoculated quinoa (control) and quinoa inoculated with arbuscular mycorrhizal fungi (AMF) and/or amended with compost (Comp) subjected to different water regimes (WW; well-watered or DS: drought stress) after 4 months of cultivation.

		Na^+^	Ca^2+^	K^+^	P
		(mg/g DM)	(mg/g DM)	(mg/g DM)	(mg/g DM)
WW	Control	0.46 ± 0.001^e^	7.59 ± 0.03^c^	3.90 ± 0.50^g^	0.02 ± 0.002^e^
	AMF	0.46 ± 0.001^f^	9.65 ± 0.07^a^	11.47 ± 0.42^c^	0.32 ± 0.007^a^
	Comp	0.39 ± 0.001^g^	9.01 ± 0.02^b^	11.54 ± 0.24^b^	0.03 ± 0.005^de^
	AMF + Comp	0.28 ± 0.001^h^	9.45 ± 0.05^a^	13.60 ± 0.45^a^	0.22 ± 0.012^b^
DS	Control	1.61 ± 0.010^a^	6.35 ± 0.09^f^	2.31 ± 0.74^h^	0.01 ± 0.001^f^
	AMF	1.09 ± 0.001^c^	6.71 ± 0.01^d^	6.67 ± 0.01^f^	0.04 ± 0.003^d^
	Comp	1.14 ± 0.001^b^	6.46 ± 0.02^e^	6.71 ± 0.24^e^	0.02 ± 0.001^de^
	AMF + Comp	0.92 ± 0.001^d^	7.61 ± 0.01^c^	7.17 ± 0.18^d^	0.10 ± 0.001^c^

Na^+^, sodium; Ca^2+^, calcium: K^+^, potassium; and P, phosphorus; Values represent the mean ± standard deviation (SD; *n* = 3). Means followed by the same letters are not significantly different (*p* ≤ 0.05).

Furthermore, several studies have indicated that compost and AMF improve plant growth by absorbing immobile soil nutrients such as N and P (Baslam et al., 2014; Al-Karaki, 2016; Barje et al., 2016; Frosi et al., 2016; Raklami et al., 2019b). Colonization by AMF results in the establishment of extensive hyphal networks and the secretion of glomalin, supplying plants with water and nutrients (P, N, K^+^, Ca^2+^, and Zn), which improves soil structure and hence productivity (Ben-Laouane et al., 2020). AMF extraradical hyphae can spread ~12 cm further beyond the root system, thus providing a greatly enhanced absorption area over roots, allowing plants to absorb root-inaccessible mineral nutrients (Bonfante and Anca, 2009; Kiers et al., 2011). Li et al. (1991) revealed that >75% of P acquired by mycorrhizal plants could be attributed to these extraradical hyphae. In addition, AMF modify root system elements, including morphology and diameter, and promote the development of a dense root system which improves plant functioning (Elsen et al., 2003). Under water-deficit conditions, plants improve in root fineness, root-shoot ratio, root number, and root hair length to mitigate the deleterious effects. Our data may suggest a modification in AMF-induced root morphology resulting in improved uptake, as observed, of mineral elements, including P, K, and *Ca*. The addition of compost to low OM soil can provide essential nutrients for plant growth and development and promote the plant’s biological activity, which can further sustain the slow release of nutrients from the organic amendment (Scotti et al., 2015). The societal implications of these results are that from a human nutrition perspective (bioavailability), quinoa plants treated with AMF provide a more nutrient-rich grain, thus contributing to greater nutrition for consumers.

### Biofertilizers Restore Abnormalities in Photosynthetic Machinery and Metabolite Content in Quinoa Grown Under Droughted-Field Conditions

The grain yield of cereals is determined by the synergistic interaction between source activity and sink (developing grains) capacity. A strong source with sufficient reserves is necessary for grain filling, and a high sink capability promotes reserve remobilization from the source to the sink (Camargo et al., 2016). We evaluated the protective role of compost-derived and rhizosphere-enriched AMF isolates application on chlorophylls as the major pigment used in photosynthesis under normal and drought conditions. Our results showed that concentration of Chl *a*, *b*, and total Chl significantly (*p* < 0.001; Supplementary Table S1) decreased under DS conditions (Table 4). One of the most important responses of plants to drought stress is the decline in concentrations of photosynthetic pigments, which may be due to a decrease in the protein, N, and Mg concentrations observed in the present study. A reduction in the chlorophyll content under water deficit suggests a breakdown of the chlorophyll structure and mechanisms of chloroplast dismantling (Rangani et al., 2016). Both Chl biosynthesis and degradation are linked with photosystem (PS) assembly and disassembly because Chl molecules are intimately integrated within PS subunits. The PS II light collection system in plants consists of several chlorophyll-binding proteins that perform essential functions, including the efficient collection of light energy for photosynthesis (Rangani et al., 2016). Concentrations of chlorophylls decreased in untreated and treated plants when subjected to drought. Concentrations of all pigments were significantly higher in treated plants than untreated plants. Applying biofertilizers induced a significant increase in the photosynthetic pigments as compared with non-inoculated with AMF and non-amended with compost. Indeed, the AMF and compost bicombination showed the highest values of these parameters under WW conditions. When water deficit was applied, all the biofertilizer treatments could maintain higher chlorophyll content than non-amended and non-inoculated control plants. A higher photosynthetic pigment under drought stress conditions suggests a better performance of the photosynthetic apparatus (Anli et al., 2020a; Ben-Laouane et al., 2020). Similar results were found in maize (Quiroga et al., 2017), quinoa (Ramzani et al., 2017), and date palm (Anli et al., 2020a). Hashem et al. (2019) showed that organic amendment and AMF mitigated the abiotic stress-induced decline in photosynthesis by improving chlorophyll biosynthesis. Several molecular and biochemical mechanisms have been proposed to explain the enhanced performance upon drought mediated by AM symbiosis and/or compost in the host plant (Fileccia et al., 2017; Balestrini et al., 2020).

**Table 4:** Leaf chlorophyll content of non-amended and non-inoculated quinoa (control) and quinoa inoculated with arbuscular mycorrhizal fungi (AMF) and/or amended with compost (Comp) subjected to different water regimes (WW; well-watered or DS: drought stress) after 4 months of cultivation.

		Chlorophyll *a*	Chlorophyll *b*	Total chlorophyll
		(mg/g DM)	(mg/g DM)	(mg/g DM)
WW	Control	5.75 ± 0.09^e^	0.69 ± 0.05^d^	6.45 ± 0.03^e^
	AMF	9.03 ± 0.01^b^	1.61 ± 0.00^b^	10.65 ± 0.01^b^
	Comp	8.48 ± 0.41^c^	1.68 ± 0.14^a^	10.17 ± 0.55^c^
	AMF + Comp	10.84 ± 0.10^a^	1.83 ± 0.19^a^	12.68 ± 0.29^a^
DS	Control	3.87 ± 0.17^f^	0.54 ± 0.04^e^	4.41 ± 0.22^f^
	AMF	6.23 ± 0.05^d^	1.13 ± 0.06^c^	7.36 ± 0.12^d^
	Comp	6.46 ± 0.10^d^	1.23 ± 0.12^c^	7.69 ± 0.02^d^
	AMF + Comp	6.40 ± 0.15^d^	1.30 ± 0.18^c^	7.70 ± 0.03^c^

Values represent the mean ± standard deviation (SD; *n* = 3). Means followed by the same letters are not significantly different (*p* ≤ 0.05).

**Figure 2:**
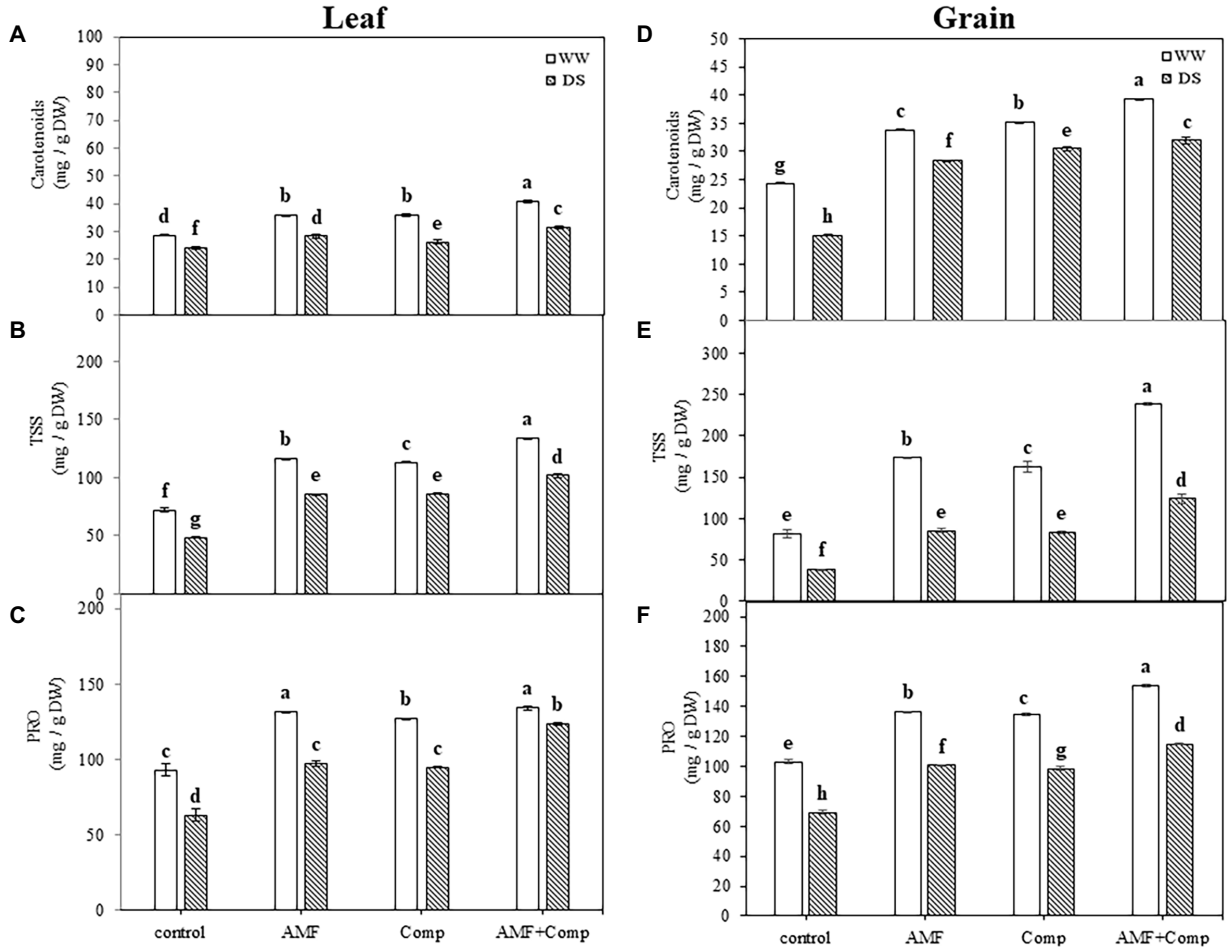
Effects of water regimes (WW; well-watered or DS: drought stress) on leaves and seeds **(A,D)** carotenoids, **(B,E)** total soluble sugar content, and **(C,F)** protein content of non-amended and non-inoculated quinoa (control), and quinoa inoculated with arbuscular mycorrhizal fungi (AMF) and/or amended with compost (Comp). Data are mean ± SD. Means followed by the same letters are not significantly different at *p* < 0.05.

Since pseudocereals, including quinoa, are broadleaf plants with seeds characterized by excellent nutrient profiles that can be ground into flour, providing a gluten-free alternative to regular cereal-based flour; we next sought to analyze the carotenoids—can be not only provitamin A but also strong antioxidants with various health-promoting properties—soluble sugars, and proteins in the leaves (source) and seeds (sink). Water deficit resulted in a significant decrease in carotenoid (32 and 15%), sugar (52 and 33%), and protein (33 and 32%) concentrations (*p* < 0.001; Supplementary Table S1) in quinoa grains and leaves, respectively, as compared to non-stressed untreated plants (Figure 2). The supply of biofertilizers induced a significant increase in these parameters compared to untreated plants regardless of the water regime. Under drought stress, the most significant increases (220 and 111%) were recorded for TSS content in grains and leaves, respectively, in plants treated with dual AMF and compost application. Quiroga et al. found similar results regarding carotenoid content in maize (Quiroga et al., 2017). The synergistic effect of AMF and compost in improving the total content of carotenoids might be due, at least partially, to nutrient availability (Fileccia et al., 2017). Tang et al. (2015, 2016) suggested that quinoa seeds consumption over other cereals is a way to increase the intake of carotenoids. Copetta et al. (2011) observed that the use of AMF and compost improved the quality of plants by improving their organic (sugar and protein) osmolyte composition through increasing the photosynthetic machinery. The increase in protein and sugar concentration could be related to the increased mineral release from the compost and the improvement of its uptake by AMF, resulting in high phytohormone levels, thereby boosting the photosynthetic apparatus and source-sink dynamics. Previous studies showed that sugar metabolism genes tend to be upregulated in plants treated with beneficial microbes under abiotic stresses (Ahanger et al., 2014; Bárzana et al., 2015). Studies have revealed that enhancement of the accumulation of osmolytes, including proteins and sugars, is regulated through modulations in their assimilatory pathways with up- and downregulation of their synthesis and catabolism (Wu et al., 2017). Begum et al. (2019) suggested that the AMF inoculation of drought-stressed plants may have improved synthesis and downregulated the catabolism of osmolytes, resulting in significant increases in their accumulation. Ahanger et al. (2018) reported that crops exhibiting higher accumulation of osmolytes show improved tolerance and growth performance under stress through tissue water content and protein structure maintenance and functioning. Osmolyte accumulation is considered a ubiquitous response for accelerating water uptake under drought conditions, and the biofertilizer-mediated enhancement in their accumulation shown *herein* justifies the beneficial role of AMF and compost in improving the grain performance of quinoa under water-deficit conditions. Keunen et al. (2013) showed that sugars are key ROS scavengers and are assumed to regulate the interplay between ROS signaling and stress tolerance.

### Biofertilizer Application Rescues Oxidative Stress Levels and Antioxidative Enzyme Activity in Quinoa Grains and Leaves

**Figure 3:**
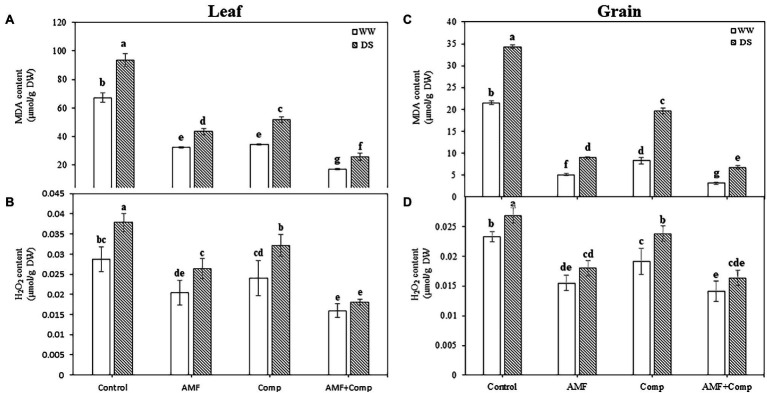
Effects of water regimes (WW; well-watered or DS: drought stress) on leaves and seeds **(A,C)** lipid peroxidation (MDA) and **(B,D)** hydrogen peroxide (H_2_O_2_) content in seeds of non-amended and non-inoculated quinoa (control), and quinoa inoculated with arbuscular mycorrhizal fungi (AMF) and/or amended with compost (Comp). Data are mean ± SD. Means followed by the same letters are not significantly different at *p* < 0.05.

Drought stress and biofertilizers significantly (*p* < 0.001, Supplementary Table S1) affected MDA and H_2_O_2_ concentrations in quinoa leaves and grains (Figure 3). MDA and H_2_O_2_ concentrations were markedly increased in leaves and grains in non-treated plants, especially under DS conditions. Drought-induced oxidative damage resulting from increased ROS, including H_2_O_2_, leads to lipid peroxidation and membrane dysfunction, thus affecting normal cellular functioning. The H_2_O_2_ generated at various sites in different plant organelles may diffuse through membranes, causing damage over long distances. Increased membrane damage, lipid peroxidation, in crops due to excessive generation of ROS has been well documented. Drought influences plant growth transitions by causing a surge in H_2_O_2_ accumulation and impeding membrane integrity (Singh et al., 2016). Stresses upregulate lipoxygenase activity and alter the poly-unsaturated fatty acid composition, leading to disruption in membranes’ structural and functional integrity (Nahar et al., 2016). Lipid peroxidation levels in leaves and grains in the droughted cultures were decreased in the presence of AMF and/or compost. Similarly to MDA concentrations, H_2_O_2_ levels were significantly lower in the cultures inoculated with AMF and/or amended with compost than in non-AMF and compost-free plants. Quinoa inoculated with mixed AMF species and amended with compost, the dual combination, showed the lowest oxidative stress values in leaves by 72 and 52% and grains by 70 and 50% in MDA and H_2_O_2_, respectively. These could be attributed to reducing the ROS concentrations to minimal possible levels, thus protecting the major metabolic processes. A reduction in oxidative stress subsequently decreases the level of lipid peroxidation (Schützendübel and Polle, 2002), which is a process in which ROS obtain electrons from the lipid bilayer (cell membrane), leading to deleterious oxidation of most cell components. Maintaining low concentrations of ROS benefits tissues in regulating and integrating key developmental events, including cell proliferation, signaling, and programmed cell death (Neill et al., 2002; Miller et al., 2010).

**Figure 4:**
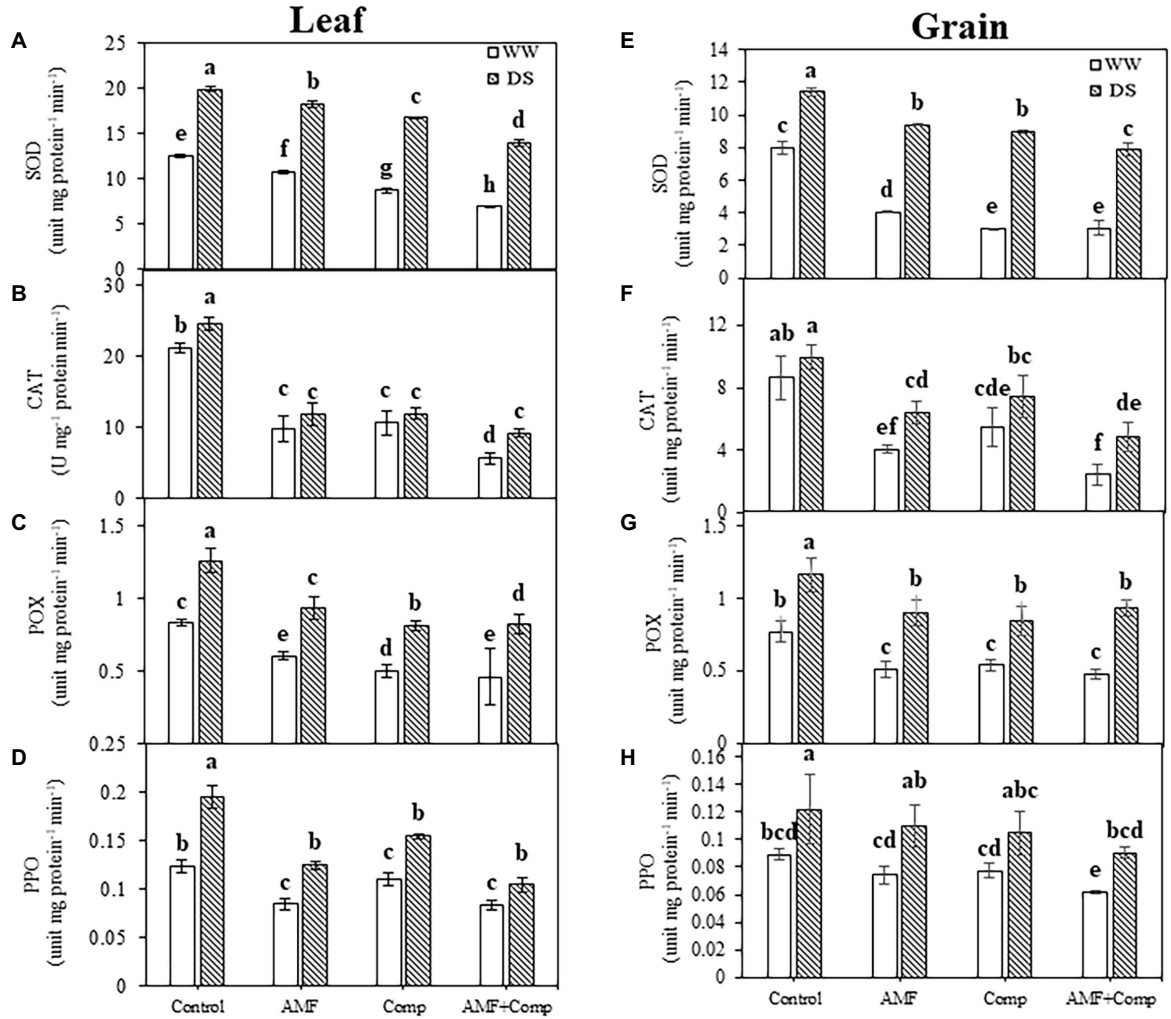
Effects of water regimes (WW; well-watered or DS: drought stress) on leaves and seeds antioxidant enzyme activity **(A,E)** superoxide dismutase (SOD), **(B,F)** catalase (CAT), **(C,G)** peroxidase (POX), and **(D,H)** polyphenol oxidase (PPO) of non-amended and non-inoculated quinoa (control), and quinoa inoculated with arbuscular mycorrhizal fungi (AMF) and/or amended with compost (Comp). Data are mean ± SD. Means followed by the same letters are not significantly different at *p* < 0.05.

Furthermore, the application of AMF and/or compost decreased the activity of the antioxidant enzymes in quinoa grains and leaves compared to untreated plants, regardless of the water regime applied. This decrease was more noticeable in the combined treatment for CAT (50 and 62%) and SOD (both 30%) activities in grains and leaves, respectively, under drought stress than in untreated plants (Figure 4). In contrast, in untreated quinoa plants, SOD, CAT, POX, and PPO activities were significantly increased (*p* < 0.001, Supplementary Table S1) under water shortage (Figure 4). The high antioxidant levels in quinoa grains and leaves grown in soil non-inoculated with AMF and non-amended by compost, independently of the water regime, may be linked to the damaging effect of the high level of H_2_O_2_ and MDA content. Several studies have shown increased antioxidant activity in drought-stressed plants, which detoxifies ROS damage and alleviates oxidative stress (Gill and Tuteja, 2010; Shahid et al., 2014; Meddich et al., 2018). The significant increase in these enzymes limits cell damage and improves the antioxidant capacity of plants to defend themselves against stress, suggesting that AMF symbiosis and/or compost could help quinoa plants reduce the oxidative damage in response to water deficiency. Increased SOD activity prevents the generation of toxic hydroxyl radicals through the Haber-Weiss reaction, which can otherwise induce severe damage to the membrane and organelle functioning. CAT and peroxidases neutralize cytosolic H_2_O_2_ excess, while other enzymes, i.e., APX, AsA, and GSH act on the AsA-GSH pathway to neutralize plastidial and mitochondrial H_2_O_2_. In drought-stressed quinoa plants, biofertilizers mediated the upregulation of antioxidant functioning and redox homeostasis maintenance more than biofertilizer-treated plants under well-watered conditions and were thus able to prevent the harmful effects of drought stress to a considerable extent.

### AMF and Compost Enhance the Bioactive Compounds of Quinoa Grains Under Droughted-Field Conditions

**Figure 5:**
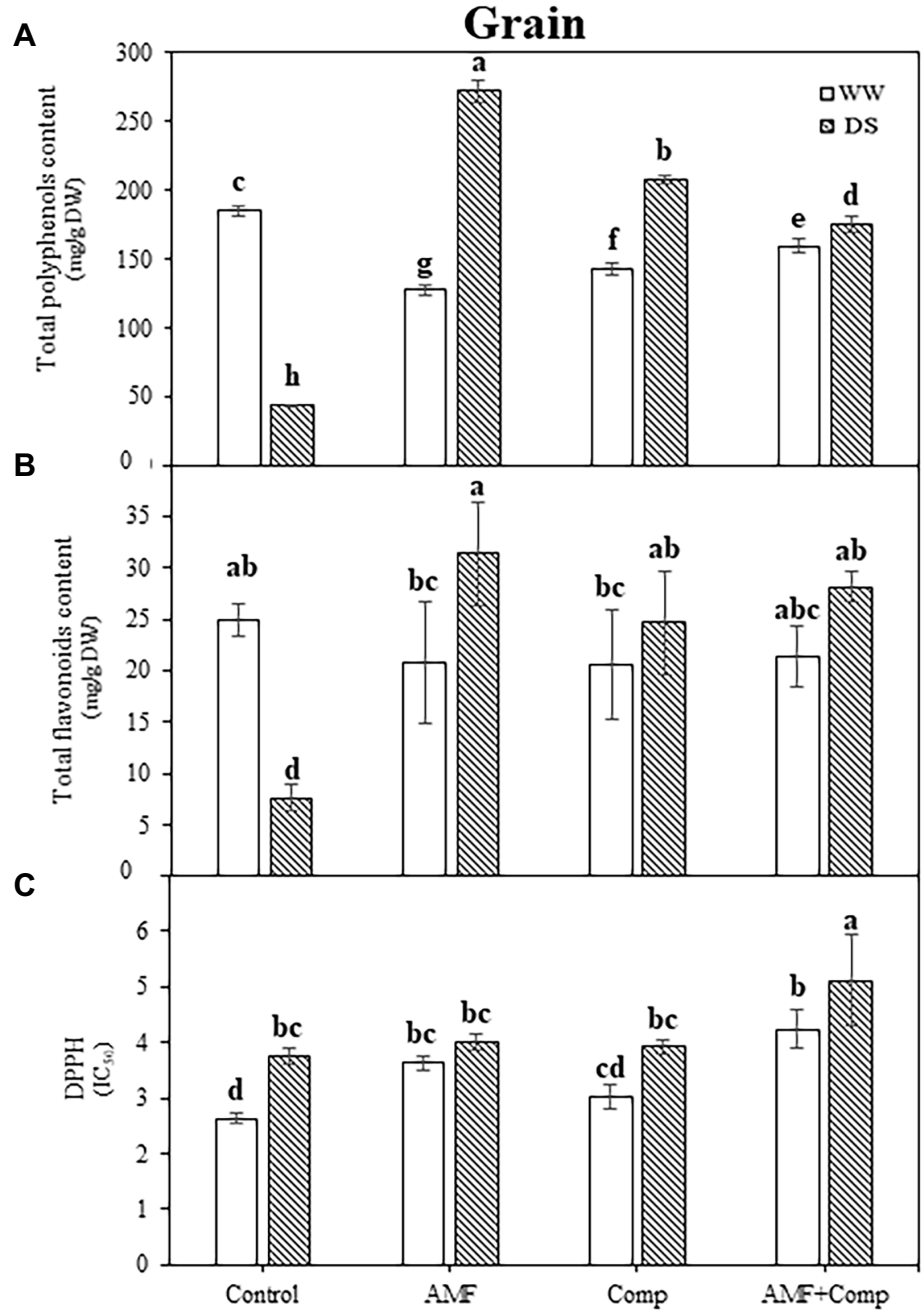
Effects of water regimes (WW; well-watered or DS: drought stress) on seed antioxidant properties **(A)** total phenolics, **(B)** total flavonoids, and **(C)** DPPH (2,2-diphenyl-1-picrylhydrazyl) of non-amended and non-inoculated quinoa (control), and quinoa inoculated with arbuscular mycorrhizal fungi (AMF) and/or amended with compost (Comp). Data are mean ± SD. Means followed by the same letters are not significantly different at *p* < 0.05.

TPC, TFC, and DPPH were progressively influenced by drought stress and biofertilizer application. In this investigation, a significant decrease in the TPC (76%) and TFC (70%) under drought stress *vs* WW was observed in quinoa grains. However, the antioxidant activity determined by the DPPH radical scavenging activity showed a significant increase (42%) in the untreated treatment (Figure 5). The application of AMF and compost, separately or in combination, increased these parameters significantly more than in stressed controls. Indeed, AMF-inoculated quinoa yielded the highest TPC improvement (525%) in grains under drought stress. It has been reported that AMF symbiosis under drought improves non-enzymatic antioxidant activities, which counteracts water-deficit effects in other cereals (Bahraminia et al., 2020). Previous studies demonstrated increased phenol and flavonoid content as drought severity increased (Gharibi et al., 2016; Siracusa et al., 2017). In plant tissues under control conditions, Robbins showed that phenylalanine produced widely dispersed phenolic acids, i.e., hydroxycinnamic acids (Robbins, 2003). The most common forms of flavonoids are glycoside derivatives, even though these compounds occasionally occur as a glycone in plants (Sarker and Oba, 2018). Flavonoids represent 60% of total dietary phenolic compounds, with the most predominant flavonoids being flavonols and the most prevalent naturally occurring flavonols being the glycosides of quercetin (Harborne and Williams, 2000). Moreover, our observation provides supportive evidence for a similar result for cereal grains (Adom and Liu, 2002; Ma et al., 2016; Kiani et al., 2021). As observed in this study, the induction of the synthesis of phenolic compounds by mycorrhization has also been shown by Hazzoumi et al. (2015), thus providing evidence of an active secondary metabolism. The increase in phenolic metabolism in response to mycorrhization would be responsible for the resistance of mycorrhizal plants to drought. The plants would respond more quickly and produce large quantities of phenolic compounds that can intervene in the defense reactions. Furthermore, phenols and flavonoids may be involved as signal molecules in plant-AMF interactions (Morandi et al., 1984), where some new phenolic molecules are produced during the establishment of AMF colonization (Charitha Devi and Reddy, 2002). Interestingly, Fiasconaro et al. reported that compost-treated soil increased TPC in peppers grown under drought conditions (Fiasconaro et al., 2019).

The high antioxidant activity induced by DPPH activity suggested the accumulation of phenolic compounds, which play an essential role in scavenging ROS under stressful conditions (Gengmao et al., 2015). Furthermore, high levels of phenols in plants can markedly improve their resilience during exposure to water stress and lead to better adaptation and survival (Liu et al., 2011). This accumulation of the essential non-enzymatic antioxidants, TPC and TFC, provides further evidence that phenol and flavonoid compounds play vital physiological and biochemical roles in cells, particularly in ROS scavenging and in helping overcome stresses (Tohidi et al., 2017; Sharma et al., 2019), and in particular, those evoked by oxidative stress during drought stress (Hura et al., 2007). From a societal and human nutrition perspective, the increase of these bioactive compounds in biofertilizer-treated quinoa grains could be very useful in preventing various diseases, including cancer and cardiovascular disease. Thus, the “empowerment of these compounds in plants, especially in grains, can limit human diseases (Uttara et al., 2009) and take the food industry to another level of quality/functionality.”

### Biofertilizers Improved Soil Fertility Traits in Post-harvest Agricultural Soil

The results of soil analyses after the cultivation of quinoa and the application of the different treatments are shown in Table 5. Compared to the initial properties (Table 1), the soil TOC, OM, and P were significantly improved after biofertilizer application (*p* < 0.01, Supplementary Table S1). pH values decreased after the experiment in quinoa plants treated with AMF + Comp reaching a pH value of 7.7 under stressed conditions. The dual application of biofertilizers significantly improved the concentrations of OM and TOC by *ca*. 200% and AP by ~50% under droughted-field conditions. The improved soil physicochemical properties after harvest under water deficit and biofertilizer application, especially compost, could be due to the adequate and balanced quantity of nutrients and OM provided by this amendment and the processes launched by chemical and biological activity. Montiel-Rozas et al. showed that the application of organic amendments soil microbial activity, increasing OM degradation and mineralization processes (Montiel-Rozas et al., 2016). Similarly, Gaiotti et al. have highlighted that the inoculation with microorganisms and application of compost effectively improved soil quality, especially OM and mineral nutrition (Gaiotti et al., 2017). Moreover, AMF could promote soil quality *via* multiple mechanisms such as P solubilization, soil structure and aggregates *via* glomalin production (Ben-Laouane et al., 2020, 2021). Biofertilizers are not fertilizers in themselves but potentially enhance the release of naturally reserved nutrients in the soil (Schütz et al., 2018). Pii et al. (2015) showed that microbial inoculants enhance the decomposition process fostering the release of essential plant nutrients produced from the natural process of OM decomposition and which induce overall crop productivity. Thus, the application of nutrient-rich biofertilizers made from organic amendments and microorganisms that can induce such phenomena as P solubilization and mineral absorption is essential in the recovery of soil nutrients under water deficit and to enhance plant life growth and yield. Therefore, in this study, scaling up the biofertilizer formulation and application in field trial provided optimum growth conditions for maintaining the viability of the microorganisms, under unfavorable conditions, and added to the compost carrier to ensure that the nutrients are made available to the plant. Thus, the need to use a cheap and readily available organic matter coupled with indigenous microorganism strains should be an integral component of agricultural practice and industrial methods to enhance soil quality and essential nutrient content, due to the direct and indirect effects, and hence to increase yield performance and stimulate the concentration and distribution of nutrients in quinoa.

**Table 5:** Main characteristics of different treatments on agricultural soil physicochemical parameters after harvest.

		pH	EC	TOC	OM	P
			(mS/cm^2^)	(%)	(%)	(ppm)
WW	Control	7.99 ± 0.08^a^	0.74 ± 0.01^d^	1.14 ± 0.05^d^	1.97 ± 0.09^d^	29.98 ± 1.65^c^
	AMF	7.91 ± 0.07^ab^	0.89 ± 0.01^bc^	2.14 ± 0.02^c^	3.70 ± 0.04^c^	33.70 ± 3.67^c^
	Comp	7.88 ± 0.11^ab^	0.80 ± 0.00^cd^	2.67 ± 0.07^b^	4.60 ± 0.21^b^	36.62 ± 2.10^b^
	AMF + Comp	7.81 ± 0.00^ab^	0.76 ± 1.35^d^	2.94 ± 0.02^a^	5.07 ± 0.04^a^	44.32 ± 3.59^a^
DS	Control	8.02 ± 0.07^a^	0.74 ± 0.01^d^	1.00 ± 0.08^e^	1.74 ± 0.14^e^	29.72 ± 4.86^c^
	AMF	7.93 ± 0.04^ab^	0.93 ± 0.04^b^	2.11 ± 0.11^c^	3.64 ± 0.20^c^	30.52 ± 4.66^c^
	Comp	7.95 ± 0.06^ab^	0.96 ± 0.01^b^	2.65 ± 0.10^b^	4.57 ± 0.17^b^	35.29 ± 3.59^b^
	AMF + Comp	7.71 ± 0.16^ab^	1.16 ± 0.15^a^	3.02 ± 0.06^a^	5.22 ± 0.11^a^	43.78 ± 3.64^a^

EC, electric conductivity; TOC, total organic content; OM, organic matter; and P, available phosphorus. Values represent the mean ± standard deviation (SD; *n* = 3). Means followed by the same letters are not significantly different (*p* ≤ 0.05).

### Principal Component Analysis and Heat Map Revealed the Potential of Biofertilizer Applications to Mitigate Drought and Support Better Quinoa Grains

**Figure 6:**
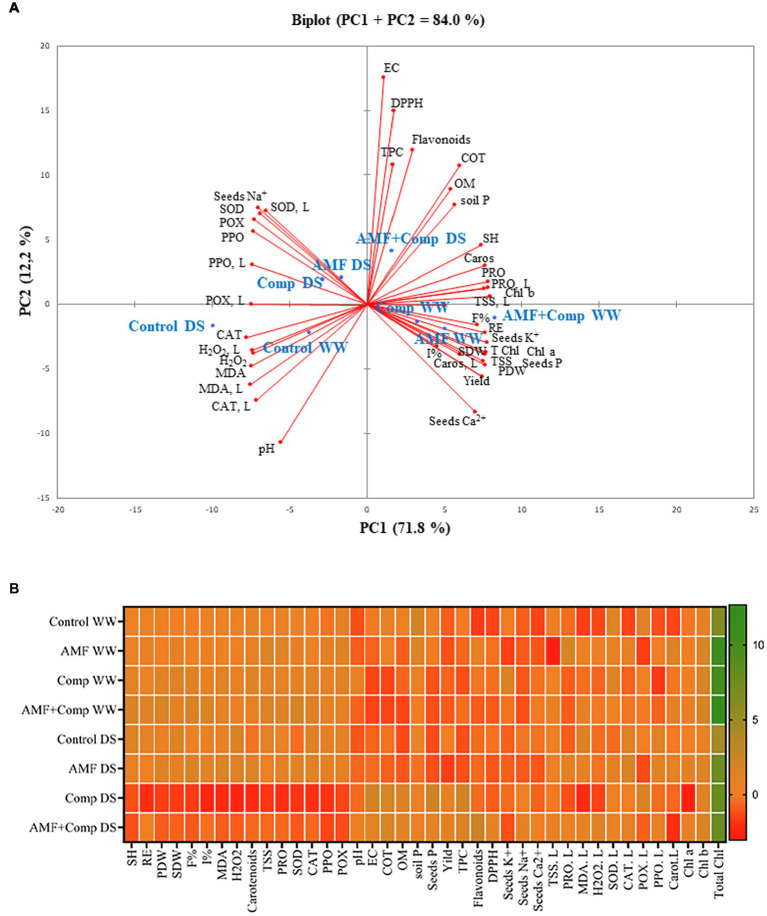
The principal component analysis (PCA) **(A)** and heat map **(B)** measured variables and applied treatments. AMF, arbuscular mycorrhizal fungi consortium; AP, soil available phosphorous; Ca^2+^, seed calcium; Caros, seed corotenoid; Caros, L; leaf carotenoid; CAT, seed catalase activity; CAT, L, leaf catalase activity; Chl, leaf chorophyll; Comp, compost; DS, drought stress; EC, electrical conductivity; F%, AMF colonization frequency; I%, AMF colonization intensity; K^+^, seed potassium; Na^+^, seed sodium; OM, organic matter; P, seed phosphorus; PDW, plant dry weight; PPO, seed polyphenol oxidase activity; PPO, L, leaf PPO; POX, seed peroxidase activity; POX, L, leaf POX; Prot, seed protein; Prot, L, leaf protein; RE, root elongation; SDW, seed dry weight; SH, shoot height; SOD, seed superoxide dismutase activity; SOD, L, leaf SOD; TFC, seed total flavonoid; TOC, total organic carbon; TPC, seeds total phenol; TSS, seed total soluble sugar; TSS, L, leaf total soluble sugar; and WW, well-watered.

The principle component analysis (PCA; Figure 6A) and heat map (Figure 6B) were performed to elaborate on the interactions across the different treatments applied and traits. The PCA shows that treatments and variables were associated (83%) with the first (PC1) and the second (PC2) components, of which PC1 was the major component (71%; Figure 6A and Supplementary Table S2). The heat map analysis indicates that the highest values of soil fertility (TOC, OM, and AP), plant growth (PH, RE, PDW, and SDW), yield, and grain traits (K, TSS, protein, and carotenoids) were found in AMF + Comp-treated quinoa. DPPH radical scavenging activity and EC registered the highest values in the dual inoculation and compost under DS conditions. The single application of AMF was correlated with F%, I%, and grain P and Ca, under WW conditions, and with grain TPC and TFC under stressful conditions. Regarding irrigation, the drought stress conditions were associated mainly with low nutrient and bioactive compound content in grains, oxidative damage, and low soil fertility, leading to reduced grain quality and yield. In fact, the lowest values for these parameters were recorded in the controls under drought conditions, which showed the highest accumulation of Na^+^, oxidative stress markers (H_2_O_2_ and MDA), and detoxifying enzymes (SOD, CAT, PPO, and POX).

In general, our study demonstrates that the combination of AMF + Comp was the most efficient treatment to improve the yield and performance parameters of quinoa grains, principally regarding the concentration of the non-enzymatic antioxidants of phenolic and flavonoid compounds, under drought stress. Moreover, under the same condition, the dual combination improved plant growth, yields, and grain quality seemed to be influenced by the increase in grain nutrient status (P, K, and Ca^2+^).

## Conclusion

Drought is the most important environmental stress factor that significantly reduces plant growth, biochemical processes, and crop production and nutritive quality worldwide. Taken together, our data suggest that drought stress limited quinoa growth, yield, and grain nutritional status/bioactive compounds and increased grain stress indicators. The overall conclusion of this study is that reinforcing the root system and altering the microbial population around the plant’s rhizosphere, thereby disturbing the biological activities of the soils’ ecosystem, increase the soil’s traits, and in turn, quinoa yield/productivity and grain quality under normal and droughted-field conditions. AMF and the provision of green compost have an influential role in improving and preserving quinoa grain yield and functionality under water shortage conditions by boosting their bioactive compounds, including flavonoid and phenolic compound concentrations, ultimately improving the beneficial health effects for the final consumer. The potential of such native AMF + compost-based fertilizer applications could help plant growth, performance, and grain biofortification and mitigate global drought—a global mitigation strategy that farmers could implement as it is eco-friendly, cost-effective, easy to apply, and precludes the need for further subsidies.

Altogether, our results suggest that using a locally produced compost and water stress-adapted endogenous AMF can benefit the vegetative growth of crops and the quality of the grain. This is an important finding since grain biofortification under water limitation is one of the main effects of climate change in agricultural areas worldwide, where the use of efficient biotechnological tools can partially counter the deleterious effects of drought. In the application of industrial biotechnology, it is more attractive and more promising to reveal the powerful features of natural consortia and organic amendments and combine to execute new functions. This knowledge is crucial to boost grain quality and bioactive compounds at the industrial and pharmaceutical levels and has the potential for extensive application with all the subsequent benefits for humanity.

## Data Availability Statement

The original contributions presented in the study are included in the article/Supplementary Material, further inquiries can be directed to the corresponding authors.

## Author Contributions

AM and MB designed and supervised the research. ST performed the experiments and carried out the analysis. MA-E-M, AB, MA, YA-R, and WB contributed to the analytic tools. ST, MA-E-M, and MB performed the data analysis and interpretation. HB-A and TM contributed to the conception and design of the work. MB and ST wrote the manuscript. MB and MA-E-M revised and finalized the manuscript. All authors read, commented on, and approved the manuscript.

## Funding

The present study was supported by the Tuniso-Moroccan Mixed Laboratories (LMTM) of Plant Physiology and Biotechnology and Climate Change LPBV2C and FOSC project (Sus-Agri-CC) from the European Union’s Horizon 2020 research and innovation program under grant agreement N862555. This work was also funded by Grant-in-Aid for Early-Career Scientists to MB (JSPS KAKENHI grant number 20K15425) and Grant for Promotion of KAAB Projects (Niigata University).

## Conflict of Interest

The authors declare that the research was conducted in the absence of any commercial or financial relationships that could be construed as a potential conflict of interest.

## Publisher’s Note

All claims expressed in this article are solely those of the authors and do not necessarily represent those of their affiliated organizations, or those of the publisher, the editors and the reviewers. Any product that may be evaluated in this article, or claim that may be made by its manufacturer, is not guaranteed or endorsed by the publisher.

## Footnotes

^1^ http://www.fao.org/quinoa-2013/en/

## Supplementary Material

The Supplementaryfor this article can be found online at: https://www.frontiersin.org/articles/10.3389/fpls.2022.860484/full#supplementary-material
