# Supplementary material for: Root Reinforcement Improved Performance, Productivity, and Grain Bioactive Quality of Field-Droughted Quinoa (Chenopodium quinoa)
Source: Front Plant Sci. 2022 Mar 18;13:860484. doi: 10.3389/fpls.2022.860484 (PMC8971987; doi:10.3389/fpls.2022.860484)
Supplement: Supplementary file 1 [file Data_Sheet_1.docx]

**Supporting tables and figures**

# Root reinforcement improved performance, productivity, and grain bioactive quality of field-droughted quinoa (*Chenopodium quinoa*)

Salma Toubali^1,2,3^, Mohamed Ait-El-Mokhtar^1,2^, Abderrahim Boutasknit^1,2,3^, Mohamed Anli^1,2,3^, Youssef Ait-Rahou^1,2^, Wissal benaffari^1,2,3^, Hela Ben-Ahmed^3^, Toshiaki Mitsui^4^, Marouane Baslam^4*^, Abdelilah Meddich^1,2,3*^

^1^ Center of Agrobiotechnology and Bioengineering, Research Unit labelled CNRST (Centre AgroBiotech-URL-CNRST-05), Physiology of abiotic stresses" team, Cadi Ayyad University, Marrakech, 40000, Morocco,

^2^ Laboratory of Agro-Food, Biotechnologies and Valorization of Plant Bioresources (AGROBIOVAL), Faculty of Science Semlalia, Cadi Ayyad University, P.O. Box 2390, Marrakesh, Morocco

^3^ Laboratoire Mixte Tuniso-Marocain (LMTM) de Physiologie et Biotechnologie Végétales et Changements Climatiques LPBV2C, Tunis 1000, Tunisia

^4^ Laboratory of Biochemistry, Faculty of Agriculture, Niigata University, Niigata, Japan

**Supporting Table 1.** ANOVA summary outputs for the independent variables.

| **Measured traits** | **D** | **M** | **C** | **DxM** | **DxC** | **MxC** | **DxMxC** |
| --- | --- | --- | --- | --- | --- | --- | --- |
| **F%** | *** | *** | *** | ** | * | *** | *** |
| **I%** | *** | *** | *** | *** | *** | *** | *** |
| **SH** | *** | *** | *** | ns | ns | *** | *** |
| **RE** | *** | *** | *** | *** | *** | ** | *** |
| **PDW** | *** | *** | *** | *** | *** | ** | ** |
| **GDW** | *** | *** | *** | ** | *** | ns | * |
| **Yield** | *** | *** | *** | ** | *** | ns | * |
| **P** | *** | *** | ns | *** | *** | *** | *** |
| **K^+^** | *** | *** | *** | *** | *** | *** | *** |
| **Na^+^** | *** | *** | *** | *** | *** | *** | *** |
| **Ca^2+^** | *** | *** | *** | *** | * | *** | *** |
| **Grain TSS** | *** | *** | *** | *** | *** | ** | ns |
| **Leaves TSS** | *** | *** | *** | *** | * | *** | ns |
| **Grain Prot** | *** | *** | *** | ** | * | *** | ns |
| **Leaves Prot** | *** | *** | *** | *** | *** | *** | *** |
| **Grain Carot** | *** | *** | *** | * | *** | *** | *** |
| **Leaves Carot** | *** | *** | *** | ** | *** | ns | ** |
| **Grain MDA** | *** | *** | *** | *** | * | *** | ns |
| **Leaves MDA** | *** | *** | *** | *** | * | *** | ns |
| **Grain H_2_O_2_** | *** | *** | *** | ns | ns | ns | ns |
| **Leaves H_2_O_2_** | *** | *** | *** | ns | ns | ns | ns |
| **TPC** | *** | *** | *** | *** | *** | *** | *** |
| **TFC** | ns | ** | ns | ** | ns | ns | ** |
| **DPPH** | *** | *** | *** | ns | ns | * | ns |
| **Grain SOD** | *** | *** | *** | ns | *** | *** | *** |
| **Leaves SOD** | *** | *** | *** | * | ns | * | * |
| **Grain CAT** | *** | *** | *** | ns | ns | ns | ns |
| **Leaves CAT** | *** | *** | *** | ns | ns | *** | ns |
| **Grain POX** | *** | *** | *** | ns | ns | *** | ns |
| **Leaves POX** | *** | *** | *** | ns | ns | ** | ns |
| **Grain PPO** | *** | * | ** | ns | ns | ns | ns |
| **Leaves PPO** | *** | *** | *** | *** | *** | *** | ns |
| **pH** | ns | ** | ** | ns | ns | ns | ns |
| **EC** | *** | *** | ** | ** | *** | ns | ns |
| **COT** | ns | *** | *** | ns | ns | *** | ns |
| **OM** | ns | *** | *** | ns | ns | *** | ns |
| **AP** | ns | ** | *** | ns | ns | ns | ns |
| **Chl a** | *** | *** | *** | *** | *** | *** | *** |
| **Chl b** | *** | *** | *** | ns | ns | *** | ns |
| **Total Chl** | *** | *** | *** | *** | *** | *** | ** |

D: Drought; C: Compost; M: arbuscular mycorrhizal fungi consortium; PDW: plant dry weight; GDW: grain dry weight; SH: shoot height; RE: root elongation; I%: AMF colonization intensity; F%: AMF colonization frequency; MDA: malondialdehyde content; TPC: grain total phenols content; TFC: grain total flavonoids content; DPPH: DPPH radical scavenging activity; TSS: grain total soluble sugar content; Prot: grain protein content; carot: grain corotenoids content; Na^+^: grain sodium content; Ca^2+^: grain calcium content: K^+^: grain potassium content; P: seeds phosphorus content; AP: soil available phosphorous concentration; SOD : superoxide dismutase activity; CAT: catalase activity; PPO: polyphenol oxidase activity; POX: peroxidase activity; EC: electrical conductivity; TOC: total organic carbon; OM: organic matter content.

**Supporting Table 2.** Loading values and percent contribution of variables on the axis identified by the principal component analysis for all treatments under drought and well-watered conditions.

|  | **Contribution of variables (%)** | | **Square cosines of variables** | |
| --- | --- | --- | --- | --- |
|  | **PC1** | **PC2** | **PC1** | **PC2** |
| **SH** | 2.957 | 1.155 | **0.849** | 0.056 |
| **RE** | 2.784 | 0.144 | **0.800** | 0.007 |
| **PDW** | 3.069 | 1.056 | **0.881** | 0.051 |
| **SDW** | 3.043 | 1.722 | **0.874** | 0.084 |
| **F%** | 1.377 | 0.000 | 0.396 | 0.000 |
| **I%** | 1.108 | 0.587 | 0.318 | 0.029 |
| **Grain MDA** | 3.038 | 1.237 | **0.872** | 0.060 |
| **Leaves MDA** | 3.108 | 2.105 | **0.893** | 0.102 |
| **Grain H_2_O_2_** | 2.961 | 0.777 | **0.850** | 0.038 |
| **Leaves H_2_O_2_** | 3.011 | 0.703 | **0.865** | 0.034 |
| **Grain Carot** | 3.197 | 0.488 | **0.918** | 0.024 |
| **Leaves Carot** | 3.156 | 1.227 | **0.906** | 0.060 |
| **Grain TSS** | 3.176 | 0.826 | **0.912** | 0.040 |
| **Leaves TSS** | 3.437 | 0.023 | **0.987** | 0.001 |
| **Grain Prot** | 3.348 | 0.167 | **0.961** | 0.008 |
| **Leaves Prot** | 3.317 | 0.089 | **0.953** | 0.004 |
| **Grain SOD** | 2.874 | 2.344 | **0.825** | 0.114 |
| **Leaves SOD** | 2.608 | 2.667 | **0.749** | 0.130 |
| **Grain CAT** | 3.317 | 0.371 | **0.952** | 0.018 |
| **Leaves CAT** | 2.810 | 3.011 | **0.807** | 0.147 |
| **Grain PPO** | 2.942 | 1.709 | **0.845** | 0.083 |
| **Leaves PPO** | 3.011 | 0.500 | **0.865** | 0.024 |
| **Grain POX** | 2.692 | 3.013 | **0.773** | 0.147 |
| **Leaves POX** | 3.051 | 0.000 | **0.876** | 0.000 |
| **pH** | 1.685 | 6.255 | **0.484** | 0.305 |
| **EC** | 0.066 | 16.875 | 0.019 | **0.822** |
| **TOC** | 1.964 | 6.312 | **0.564** | 0.307 |
| **OM** | 1.599 | 4.285 | **0.459** | 0.209 |
| **AP** | 1.768 | 3.211 | **0.508** | 0.156 |
| **P** | 1.969 | 0.824 | **0.566** | 0.040 |
| **Yield** | 3.043 | 1.722 | **0.874** | 0.084 |
| **TPC** | 0.147 | 6.320 | 0.042 | 0.308 |
| **TFC** | 0.461 | 7.802 | 0.132 | 0.380 |
| **DPPH** | 0.163 | 12.258 | 0.047 | **0.597** |
| **K^+^** | 3.186 | 0.264 | **0.915** | 0.013 |
| **Na^+^** | 2.313 | 2.851 | **0.664** | 0.139 |
| **Ca^2+^** | 2.659 | 3.783 | **0.763** | 0.184 |
| **Chl a** | 3.213 | 0.766 | **0.923** | 0.037 |
| **Chl b** | 3.114 | 0.077 | **0.894** | 0.004 |
| **Total Chl** | 3.257 | 0.472 | **0.935** | 0.023 |

PDW: plant dry weight; GDW: grain dry weight; SH: shoot height; RE: root elongation; I%: AMF colonization intensity; F%: AMF colonization frequency; MDA: malondialdehyde content; TPC: grain total phenols content; TFC: grain total flavonoids content; DPPH: DPPH radical scavenging activity; TSS: grain total soluble sugar content; Prot: grain protein content; carot: grain carotenoids content; Na^+^: grain sodium content; Ca^2+^: grain calcium content: K^+^: grain potassium content; P: grain phosphorus content; AP: soil available phosphorous concentration; SOD : superoxide dismutase activity; CAT: catalase activity; PPO: polyphenol oxidase activity; POX: peroxidase activity; EC: electrical conductivity; TOC: total organic carbon; OM: organic matter content.


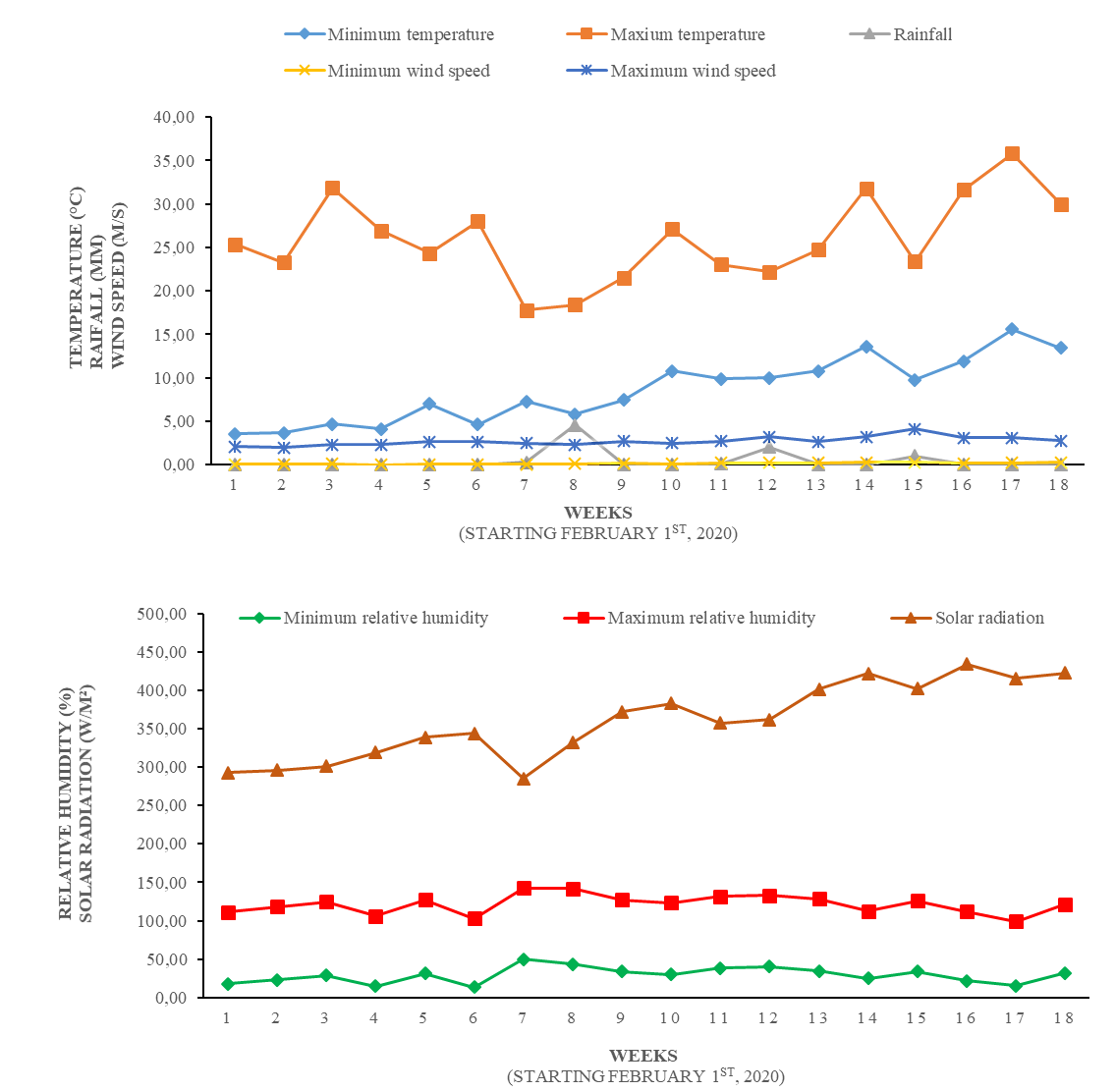


**Supporting Figure 1**. Site weather data during the experimentation period February-June 2020.
